# Impact of *Enterococcus faecium* Kimate-X on Reducing Stress in Dogs Through Gut Microbiota Modulation

**DOI:** 10.3390/vetsci12050412

**Published:** 2025-04-27

**Authors:** Rui Zhang, Wanjin Hu, Saiwei Zhong, Weiyang Chen, Meiru Chen, Qinghua Yu

**Affiliations:** 1 Laboratory of Microbiology, Immunology and Metabolism, Diprobio (Shanghai) Co., Ltd., Shanghai 200050, China; wanjin.hu@diprobio.com (W.H.); seven.zhong@diprobio.com (S.Z.); acy.chen@diprobio.com (W.C.); lina.chen@diprobio.com (M.C.); qinghua.yu@diprobio.com (Q.Y.); 2 College of Veterinary Medicine, Nanjing Agricultural University, Nanjing 210014, China

**Keywords:** stress, *Enterococcus faecium*, gut microbiota, probiotics, short-chain fatty acids

## Abstract

Stress in dog breeding leads to significant physiological and psychological burdens, including anxiety, reduced appetite, weakened immune function, gut microbiota imbalance, and even death. Currently, there are various pharmacological interventions for stress management, but few focus on gut health. This study evaluates the potential of a novel strain, *Enterococcus faecium* Kimate-X, in alleviating transport stress and improving gut health in dogs, providing an alternative to traditional pharmacological treatments. In vitro experiments showed that Kimate-X significantly enhanced the activities of superoxide dismutase (SOD) and catalase (CAT) while reducing the levels of malondialdehyde (MDA) and tumor necrosis factor-α (TNF-α) in RAW 264.7 macrophage cells. In vivo, dogs supplemented with Kimate-X exhibited significantly lower cortisol levels after transport, indicating reduced stress. Metagenomic analysis revealed increased gut microbiota diversity and higher concentrations of short-chain fatty acids (acetate, propionate, and butyrate) in fecal samples. This study systematically uncovers the mechanism by which *Enterococcus faecium* Kimate-X alleviates transport stress through modulation of the gut microbiota. These findings provide new scientific evidence supporting the use of probiotics as a novel approach to stress management in animals.

## Simple Summary

Dogs often experience stress, which can lead to a range of health problems such as anxiety, digestive issues, and weakened immunity. While there are medications to help manage stress, these drugs often come with side effects. We administered *Enterococcus faecium* Kimate-X at a concentration of 2 × 10^9^ CFU per day, given once each morning for 49 consecutive days. This study explores the potential of a new probiotic, *Enterococcus faecium* Kimate-X, as a solution to reduce stress and improve gut health in dogs. The research showed that this probiotic helps reduce stress hormones in dogs during transport and improves the balance of microbial communities in their intestines. This study offers new insight into how probiotics can support the well-being of dogs, providing an alternative to traditional treatments that focus only on symptoms. By promoting gut health, this approach can help dogs feel better and be more comfortable during stressful situations, improving their overall health and quality of life.

## 1. Introduction

In contemporary society, pets have become integral members of many households. However, with the rapid pace of urbanization and evolving lifestyles, the stressors that pets encounter have grown increasingly diverse and complex [1]. As a non-specific physiological response, stress can trigger abnormal behaviors in pets and may also lead to a range of physiological disorders, including immune suppression, metabolic imbalances, and neuroendocrine alterations [2,3]. Unfortunately, there are still limited effective strategies for the prevention and management of pet stress. In household settings, pet owners often lack the scientific knowledge and effective methods needed to address stress-related issues. Among these stressors, transport-related stress is especially common in dogs, as frequent travel—whether for veterinary visits, grooming, or relocation—can significantly elevate anxiety and physiological stress responses [4]. Pharmacological interventions, such as Gamma-Aminobutyric Acid (GABA) agonists (which enhance inhibitory neurotransmission) and Trazodone (a serotonin antagonist), are commonly prescribed for stress management in dogs. While these treatments can effectively reduce anxiety and other stress symptoms, they may have side effects such as sedation, changes in behavior, and potential dependence [5]. Consequently, there is a growing need for non-pharmacological strategies to manage stress in pets more safely and sustainably.

Recent research has increasingly highlighted the crucial role of gut microbiota in the stress response [6]. Stress can increase intestinal permeability, trigger the release of inflammatory mediators, and disrupt gut microbiota by activating the hypothalamic–pituitary–adrenal (HPA) axis and the autonomic nervous system [7]. The diversity and stability of the gut microbiota play a vital role in maintaining host health. Specifically, diversity refers to the richness and abundance of microbial species within the community, while stability denotes the ability of the microbial ecosystem to maintain its structure and function over time or under external disturbances. Diversity contributes to functional redundancy and ecological buffering capacity, whereas stability ensures the continuity of microbial functions and resilience against perturbations. The disruption of gut microbial homeostasis has been widely observed not only in humans but also in dogs. Maintaining gut microbiota diversity and stability is considered an effective strategy for mitigating stress responses, and the use of probiotics has shown potential benefits in restoring gut microbial balance, reducing inflammation, and modulating the immune system [8,9,10].

Probiotics are defined as live microorganisms that confer health benefits to the host when administered in adequate amounts [11]. In pets, probiotics have been widely used to improve digestive function and regulate immune responses [12,13,14]. However, research on the relationship between pet stress and gut microbiota remains limited, especially with regard to systematic studies on the role of probiotics. Given that the core mechanisms of the stress response involve hormone secretion and inflammatory processes, this study uses transportation as a model to induce stress in dogs, with *Enterococcus faecium* Kimate-X selected as the probiotic intervention (Kimate-X was isolated from the feces of healthy police dogs). The study aims to explore the role of Kimate-X in regulating stress-related hormone levels, alleviating inflammatory responses, and restoring gut microbiota balance, thereby elucidating its potential mechanisms in mitigating transportation stress in dogs.

## 2. Methods

### 2.1. Study on the Alleviation of Oxidative Stress by Enterococcus faecium Kimate-X In Vitro

Mouse monocyte macrophage leukemia cells (RAW 264.7 cells) were cultured in Dulbecco’s Modified Eagle Medium (DMEM) medium containing 10% Fetal Bovine Serum (FBS) and 1% antibiotics, and incubated at 37 °C in a humidified incubator with 5% CO_2_. Oxidative stress was induced in well-grown RAW 264.7 cells (cell concentration of 5 × 10^5^/mL) using 500 ng/mL LPS. Simultaneously with the induction of oxidative stress, Kimate-X was added to the culture medium containing RAW 264.7 cells at an inoculation amount of 1 × 10^5^ CFU. It is important to note that the 1 × 10^5^ CFU refers to the total bacterial count per well, rather than a concentration expressed in CFU/mL. We established three groups for the in vitro experiment using RAW 264.7 cells. (1) Control group (Control): cells were cultured under standard conditions without LPS or probiotic treatment. (2) Oxidative stress model group (LPS): cells were exposed to LPS to induce oxidative stress. (3) Probiotic treatment group (LPS + Kimate-X): cells were co-treated with LPS and *Enterococcus faecium* Kimate-X to evaluate the potential protective effects of the probiotic under oxidative stress. Each group had three replicates, which were included to enhance the reliability and reproducibility of the experimental results. After 12 h of incubation, the cell supernatants were collected, and changes in the levels of superoxide dismutase (SOD), catalase (CAT), Glutathione (GSH), malondialdehyde (MDA), Glutathione Peroxidase (GSH-Px), and TNF-α were measured using commercial ELISA kits (Meilian Biotechnology Co., Ltd., Shanghai, China).

### 2.2. Animals and Experimental Design

Sixteen Beagle dogs (male, aged 4–5 months) were grouped by body weight (BW) (*n* = 8 per group) into the basal diet (Control) group. *Enterococcus faecium* Kimate-X was prepared as a freeze-dried powder using skim milk powder as a protective agent (cryoprotectant). Each dog in the treatment group received a total of 1 g of this freeze-dried product per day, containing 2 × 10^9^ CFU of the probiotic. The Control group was fed an equivalent of 1 g of freeze-dried skim milk powder (without probiotic) under the same feeding schedule. In this study, we chose a relatively homogeneous population (juvenile male Beagle dogs) and maintained consistent dietary and housing conditions to reduce inter-individual variability. The body weight of the Control group was 5 ± 0.72 kg, and that of the Kimate-X group was 5 ± 1.21 kg, with no significant difference between the groups (*p* > 0.05). The use of juvenile male Beagle dogs was chosen to reduce variability due to age and sex, as young dogs have a more consistent immune response, which helps to minimize experimental variations. This design also limits the potential influence of sex-related differences in immune and stress responses, ensuring more consistent results.

The animal experiment consisted of two periods: the feeding period and the transport period. During the feeding period, each Beagle dog was housed in an identical dog cage (2.5 × 1.5 × 1.5 m) for 49 days, fed at 6:00 AM and 5:00 PM daily, with free access to water. No medications (e.g., antibiotics) were administered from 1 month before the study until the end of the experiment. On the 50th day, each dog was transferred to a separate cage and transported in the same vehicle. The transport route included both highways and urban roads, with a total duration of 3 h and an average speed of 60 km/h. The temperature inside the truck compartment was maintained at 25 °C with a relative humidity of 60%. Transportation began at 8:00 AM.

All experimental procedures were approved by the Animal Ethics Committee of Nanjing Agricultural University (Approval Number: NJAU. NO20231229197).

### 2.3. Blood Sample Collection and Analysis

Blood samples were collected immediately after transportation. During blood collection, one veterinarian gently restrained the dog with one arm and lightly gripped the proximal forelimb to enhance venous filling, while another veterinarian collected the blood sample. A total of 3 mL of blood was drawn from the forelimb vein using vacuum blood collection tubes without anticoagulants. The blood samples were centrifuged at 3000× *g* for 10 min at room temperature to collect serum, which was then stored at −80 °C for the subsequent measurement of stress and inflammation-related indicators. Commercial ELISA kits (Meilian Bio, Shanghai Meilian Biotechnology Co., Ltd., Shanghai, China) were used to measure cortisol, SOD, MDA and TNF-α, according to the manufacturer’s instructions.

### 2.4. Fecal Sample Collection and Analysis

After blood collection, the dogs were returned to their respective cages. Fresh fecal samples were collected from the dogs within 15 min of defecation. The samples were aliquoted and stored at −80 °C for subsequent analysis of fecal short-chain fatty acids (SCFAs) and microbiota composition.

### 2.5. DNA Extraction and Shotgun Metagenomic Sequencing

Genomic DNA was isolated from fecal samples using the QIAamp PowerFecal Pro DNA Kit (QIAGEN, Hilden, Germany) according to the manufacturer’s instructions. Two methods of QC used for DNA samples: (1) DNA degradation degree and potential contamination were monitored on 1% agarose gels; (2) DNA purity was first evaluated by measuring the 260/280 nm absorbance ratio (OD value) on a spectrophotometer to ensure it was within 1.8–2.0. The DNA concentration was then quantified using the Qubit™ dsDNA Assay Kit (Thermo Fisher Scientific, Waltham, MA, USA). Samples with a 260/280 ratio of 1.8–2.0 and a total DNA mass above 1 µg were deemed suitable for library construction.

A total amount of 1 μg DNA per sample was used as input material for library preparations. Sequencing libraries were generated using NEBNext^®^ Ultra™ DNA Library Prep Kit for Illumina (NEB, Ipswich, MA, USA) following the manufacturer’s recommendations, and index codes were added to attribute sequences to each sample. Briefly, the DNA sample was fragmented by sonication to a size of 350 bp, and then DNA fragments were end-polished, A-tailed, and ligated with the full-length adaptor for Illumina sequencing with further PCR amplification. Finally, PCR products were purified using AMPure XP beads (Beckman Coulter, Brea, CA, USA), and the resulting libraries were assessed for size distribution on an Agilent 2100 Bioanalyzer (Agilent Technologies, Santa Clara, CA, USA) and quantified by real-time PCR.

The clustering of the index-coded samples was performed on a cBot Cluster Generation System according to the manufacturer’s instructions. After cluster generation, the library preparations were sequenced on an Illumina Novaseq 6000 platform, and paired-end reads were generated.

### 2.6. Sequencing Data Processing

Briefly, Fastp (https://github.com/OpenGene/fastp (accessed on 16 June 2024), version 0.23.4) was utilized to remove the adapters and low-quality reads. Host reads were removed after alignment, reference genome database was created using bowtie2-build (https://github.com/BenLangmead/bowtie2 (accessed on 16 June 2024), version 2.5.1), including (1) dog reference genome database (Dog10K-Boxer-Tasha, NCBI accession number GCA-000002285.5); (2) human reference genome database (GRCh38.p13, NCBI accession number NC-000001.11), and obtained sequencing reads that not mapped to the reference genome using samtools (https://github.com/samtools/samtools (accessed on 16 June 2024), version 1.17). The high-quality and non-host reads were assembled using MEGAHIT (https://github.com/voutcn/megahit (accessed on 16 June 2024), version 1.2.9), and contigs with lengths greater than 500 bp were used for gene (Open Reading Frames, ORFs) prediction by Prodigal (https://github.com/hyattpd/Prodigal (accessed on 16 June 2024), version 2.6.3). Gene sequences were clustered into a non-redundant gene catalog using CD-HIT (https://github.com/weizhongli/cdhit (accessed on 16 June 2024), version 4.8.1) at 95% identity and 90% coverage. Moreover, gene abundance was estimated with BWA (https://github.com/lh3/bwa (accessed on 16 June 2024), version 0.7.17-r1198-dirty) by mapping high-quality and non-host reads to the non-redundant gene catalog.

### 2.7. Microbial Taxonomic and Functional Profiles and Analysis

Taxonomic classification was assigned to metagenomic high-quality and non-host reads with MetaPhlAn4 (https://github.com/biobakery/MetaPhlAn (accessed on 16 June 2024)) with default parameters. The read counts of species were converted into relative abundance for further analysis. The non-redundant gene was annotated with EggNOG mapper (https://github.com/eggnogdb/eggnog-mapper (accessed on 16 June 2024), version 2.1.10) based on EggNOG orthology data. The relative abundances of EggNOG genes, KEGG KO groups, or pathways were estimated by summing the relative abundances of genes annotated to belong to the same KOs or pathways.

The microbiota diversity analyses (including alpha and beta diversity) were conducted and visualized using the vegan and ggplot2 packages in R (version 4.2.1). Specifically, alpha diversity metrics, such as Shannon and Simpson indices, were calculated with the vegan package. The differences in alpha diversity indices between groups were tested by the Wilcoxon rank sum test. Principal Coordinates Analysis (PCoA) based on Bray–Curtis distances to examine the differences in the structures of the microbial communities. Differences in PCoA ordination between groups were tested by Permutational Multivariate Analysis of Variance (PERMANOVA). The discriminative microbial taxonomy between the groups was identified using linear discriminant analysis effect size (LEfSe) with a linear discriminant analysis (LDA) score > 2.0. Differential EggNOG gene KOs and pathways were identified as the same pipeline.

### 2.8. Fecal Short-Chain Fatty Acid Analysis

Fecal samples were sent to Zhongke New Life Biotechnology Co., Ltd. (Shanghai, China) for SCFA analysis. The specific method was as follows: samples were thawed on ice, and an appropriate amount was placed in a 2 mL centrifuge tube. Then, 50 μL of 20% phosphoric acid was added for resuspension, followed by the addition of 4-methylvaleric acid at a final concentration of 500 μM as an internal standard, and mixed thoroughly for 2 min. The mixture was centrifuged at 14,000× *g* for 20 min, and the supernatant was transferred to an injection vial for GC-MS analysis. A 1 μL aliquot was injected with a split ratio of 10:1. Samples were separated using an Agilent DB-FFAP capillary column (30 m × 250 μm × 0.25 μm) gas chromatography system. The temperature program was as follows: initial temperature of 90 °C; increased at 10 °C/min to 160 °C; then increased at 40 °C/min to 240 °C and held for 5 min. Helium was used as the carrier gas at a flow rate of 1.0 mL/min. A QC sample was set for every certain number of experimental samples in the sample queue to detect and evaluate the system’s stability and reproducibility.

### 2.9. Statistical Analysis

First, the Shapiro–Wilk test and Levene’s test were, respectively, used to assess the normality and homogeneity of variance of the data. For the in vitro experiments, one-way ANOVA was employed to compare differences among treatment groups; if the ANOVA result was significant, Tukey’s HSD test was used for multiple comparisons. If the data did not meet normality or homogeneity of variance, the Kruskal–Wallis test and Dunn’s test were applied. For the in vivo experiments, the primary comparison was between the Kimate-X group and the Control group. Variables meeting the assumptions of normal distribution and homogeneity of variance were analyzed using an independent-sample *t*-test, while non-normal data were analyzed using the Mann–Whitney U test. Differences in microbial diversity indices and community composition were assessed using the Wilcoxon test and PERMANOVA based on Bray–Curtis distances. All statistical analyses were performed in SPSS 26.0, figures were generated using GraphPad Prism 8.0, and microbiome analyses were conducted in R 4.2.1. All tests were two-sided, with *p* < 0.05 considered statistically significant.

## 3. Results

### 3.1. Effects of Enterococcus faecium Kimate-X on Alleviating Oxidative Stress In Vitro

**Figure 1:**
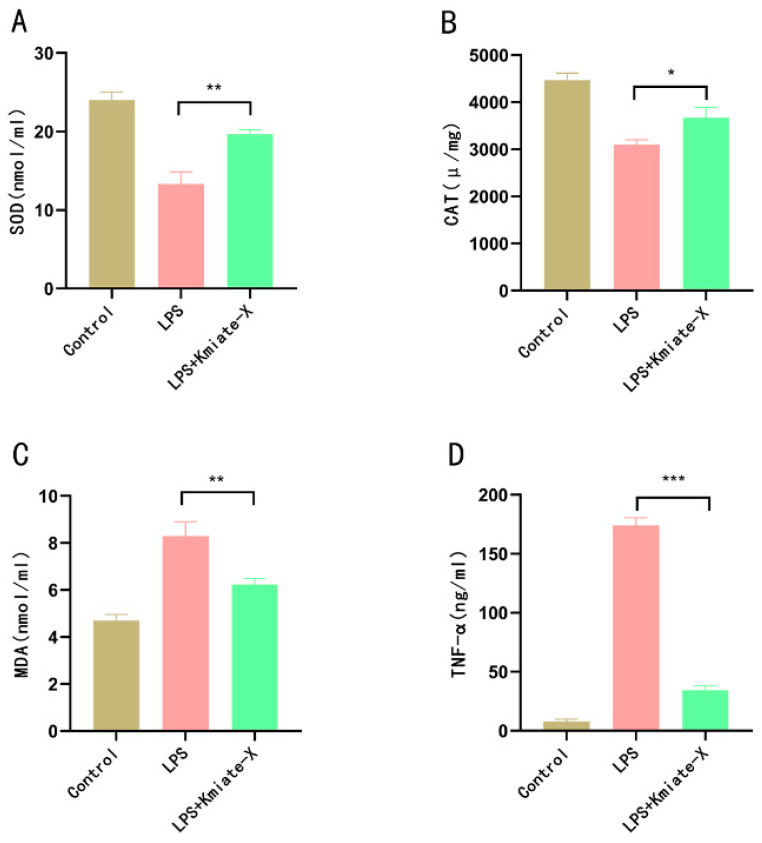
Effects of *Enterococcus faecium* Kimate-X on alleviating LPS-induced oxidative stress in RAW 264.7 cells. (**A**) superoxide dismutase (SOD), (**B**) catalase (CAT), (**C**) malondialdehyde (MDA), and (**D**) tumor necrosis factor-α (TNF-α) in the culture supernatants. Bars represent the mean ± SD of three independent experiments (n = 3). Statistical significance was determined by Tukey’s HSD test (* *p* < 0.05, ** *p* < 0.01, *** *p* < 0.001).

We used the RAW 264.7 cell model to study the effect of *Enterococcus faecium* Kimate-X on alleviating LPS-induced oxidative stress. The SOD activity in the LPS + Kimate-X treatment group was significantly higher than that in the LPS group (*p* < 0.01), but lower than that in the Control group (Figure 1A). The results showed that the CAT activity in the LPS + Kimate-X treatment group was significantly higher than that in the LPS group (*p* < 0.05) (Figure 1B). The MDA content in the LPS + Kimate-X treatment group was significantly lower than that in the LPS group (*p* < 0.01), approaching the level of the Control group (Figure 1C). The TNF-α level in the LPS + Kimate-X treatment group was significantly lower than that in the LPS group (*p* < 0.001), approaching the level of the Control group (Figure 1D).

### 3.2. Effects of Enterococcus faecium Kimate-X on Antioxidant and Inflammatory Markers in Transport-Stressed Dogs

**Figure 2:**
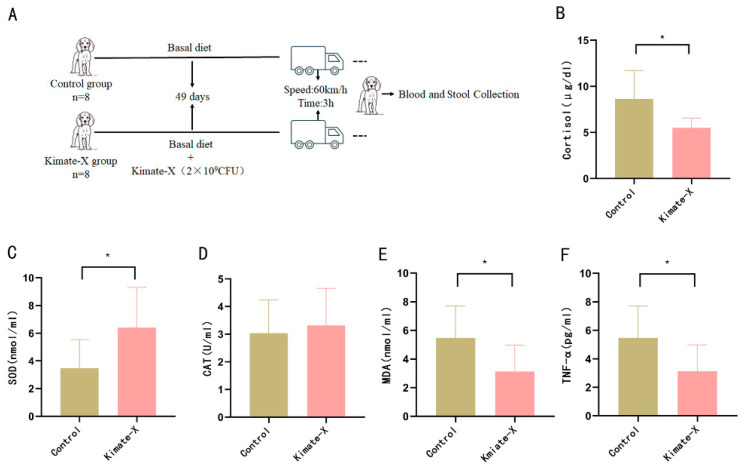
Effects of *Enterococcus faecium* Kimate-X on antioxidant and inflammatory markers in transport-stressed dogs. (**A**) Schematic overview of the experimental design. (**B**) Cortisol levels, (**C**) SOD activity, (**D**) CAT activity, (**E**) MDA content, and (**F**) TNF-α concentration are shown. Data are presented as mean ± SD (n = 8 per group). Asterisks indicate significant differences between the Control and Kimate-X groups (* *p* < 0.05), as determined by Student’s *t*-test.

Given the demonstrated antioxidant and anti-inflammatory properties of *Enterococcus faecium* Kimate-X in vitro, we evaluated its effects on oxidative stress and inflammatory responses in dogs subjected to transportation stress (Figure 2A). The results showed that, after transportation stress, serum cortisol levels in the Kimate-X group were significantly lower than those in the Control (*p* < 0.05) (Figure 2B). Additionally, SOD activity in the Kimate-X group was significantly higher than in the Control (*p* < 0.05) (Figure 2C), while there was no significant difference in CAT activity between the two groups (Figure 2D). Furthermore, MDA levels in the Kimate-X group were significantly lower than those in the Control (*p* < 0.05) (Figure 2E). These findings indicate that Kimate-X can alleviate the stress response induced by transportation. In terms of inflammatory markers, serum TNF-α levels in the Kimate-X group were significantly lower than those in the Control (*p* < 0.05) (Figure 2F). Overall, these results suggest that *Enterococcus faecium* Kimate-X can effectively mitigate the negative effects of transportation stress in dogs by enhancing antioxidant capacity and reducing inflammatory responses.

### 3.3. Results of Fecal Microbiota Composition Analysis

**Figure 3:**
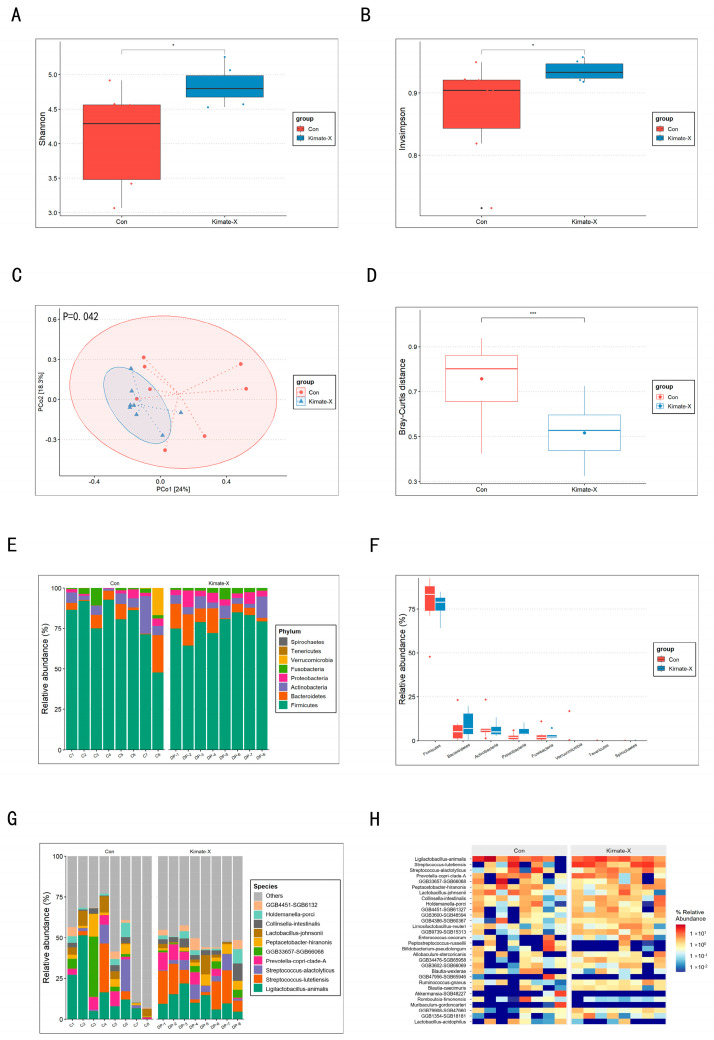
Composition of fecal microbiota in dogs based on metagenomic gene sequencing data. (**A**) Shannon index and (**B**) Simpson index were used to assess alpha diversity; (**C**) Principal Coordinates Analysis (PCoA) and (**D**) Bray–Curtis distance illustrate beta diversity. (**E**) Bar plot of relative abundances at the phylum level; (**F**) box plot comparing phylum-level relative abundances; (**G**) bar plot of selected species-level taxa; (**H**) heatmap of species-level relative abundances (rows represent individual taxa, columns represent individual dogs). Warmer colors (red) indicate higher abundance, while cooler colors (blue) indicate lower abundance. Statistical significance was evaluated by Wilcoxon rank-sum tests and PERMANOVA (* *p* < 0.05, *** *p* < 0.001).

We analyzed the fecal microbiota composition of dogs in the Control and the Kimate-X treatment group using metagenomic data. The results showed that the Shannon index and Simpson index of the Kimate-X group were significantly higher than those of the Control (*p* < 0.05) (Figure 3A,B). The PCoA plot based on the Bray–Curtis distance matrix (Figure 3C) showed clear differences in the gut microbiota structure between the Control and the Kimate-X group, with the two groups distinctly separated on the PCoA plot, indicating that Kimate-X treatment significantly altered the gut microbiota community structure. Bray–Curtis distance analysis (Figure 3D) further confirmed this, as the Bray–Curtis distance of the Control was significantly higher than that of the Kimate-X group (*p* < 0.001), demonstrating that Kimate-X treatment significantly changed the gut microbiota composition. At the phylum level, the bacterial taxa composition was similar between the Control and the Kimate-X group, but the relative abundance of major phyla differed (Figure 3E). Box plots (Figure 3F) further confirmed these observations. At the species level (Figure 3G), the relative abundance of *Prevotella copri* clade A and *Streptococcus lutetiensis* was higher in the Kimate-X group, whereas the relative abundance of *Lactobacillus johnsonii* was higher in the Control. The heatmap of gut microbiota relative abundance at the species level (Figure 3H) showed that *Prevotella copri* clade A and *Ligilactobacillus animalis* were more abundant in the Control, while *Streptococcus lutetiensis* and *Streptococcus alactolyticus* were more abundant in the Kimate-X group, indicating that these species played an important role in the Kimate-X group. Additionally, the relative abundance of *Prevotella copri* clade A and *Ligilactobacillus animalis* was lower in the Kimate-X group. Detailed information on the microbiota is provided in Supplementary Table S1.

### 3.4. Metabolic Pathways and Gene Expression in the Gut Microbiota

**Figure 4:**
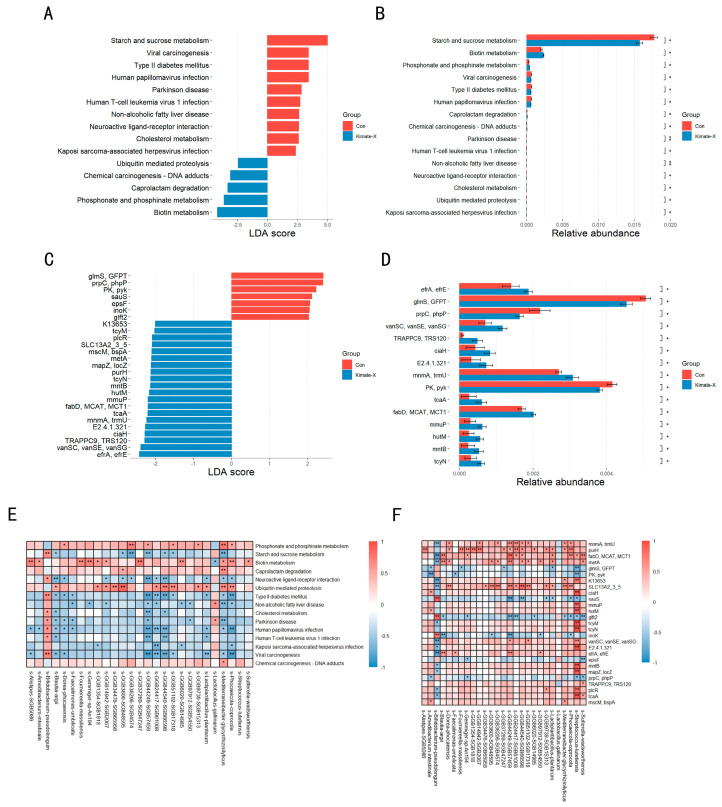
Composition, gene differences, and correlation analysis of fecal microbiota in dogs. Metagenomic data from the same fecal samples as in Figure 3 were used for functional annotation. (**A**,**C**) show LDA (linear discriminant analysis) scores from LEfSe indicating differentially abundant pathways or gene functions, whereas (**B**,**D**) display their relative abundances. (**E**) Heatmap of correlations between differential bacterial species and metabolic pathways; (**F**) heatmap of correlations between species and functional genes. A positive correlation is represented in red, and a negative correlation in blue, with deeper color intensity indicating a stronger correlation. Data were compared using the LEfSe method (Kruskal–Wallis + Wilcoxon test, followed by LDA). Differences were considered significant when the LDA score was greater than 2 and *p* < 0.05 (* indicates *p* < 0.05, and ** indicates *p* < 0.01).

The KEGG functional database pathway analysis (Figure 4A,B) revealed that the relative abundance of starch and sucrose metabolism was significantly higher in the Control compared to the Kimate-X group, while the relative abundances of biotin metabolism and phosphate and phosphonate metabolism were significantly higher in the Kimate-X group compared to the Control. The KEGG functional database KO gene analysis (Figure 4C,D) showed that the relative abundances of *efrA* and *efrE* were significantly higher in the Kimate-X group compared to the Control, whereas *glmS* and *GFPT* had higher relative abundances in the Control compared to the Kimate-X group. Additionally, the relative abundances of *vanSC*, *vanSE*, and *vanSG* were significantly higher in the Kimate-X group compared to the Control.

Correlation analysis between differential species and differential metabolic pathways (Figure 4E) found that starch and sucrose metabolism were positively correlated with several bacterial species, particularly *Bifidobacterium pseudolongum* and *Lactobacillus gallinarum*. Biotin metabolism was significantly positively correlated with *Faecalimonas umbilicata* and some unnamed bacterial species. Furthermore, pathways associated with non-alcoholic fatty liver disease and Parkinson’s disease also showed significant correlations with different bacterial species.

Correlation analysis between differential species and differential genes (Figure 4F) revealed that biotin metabolism, related to functional pathways, showed significant positive correlations with specific bacterial species, such as the significant positive correlation between *Faecalimonas umbilicata* and biotin metabolism. Additionally, some unnamed bacterial species, such as GGB3600, SGB4574, GGB3600, and SGB4859, were associated with biotin metabolism and displayed significant correlations.

### 3.5. Results of Fecal SCFA Analysis

**Figure 5:**
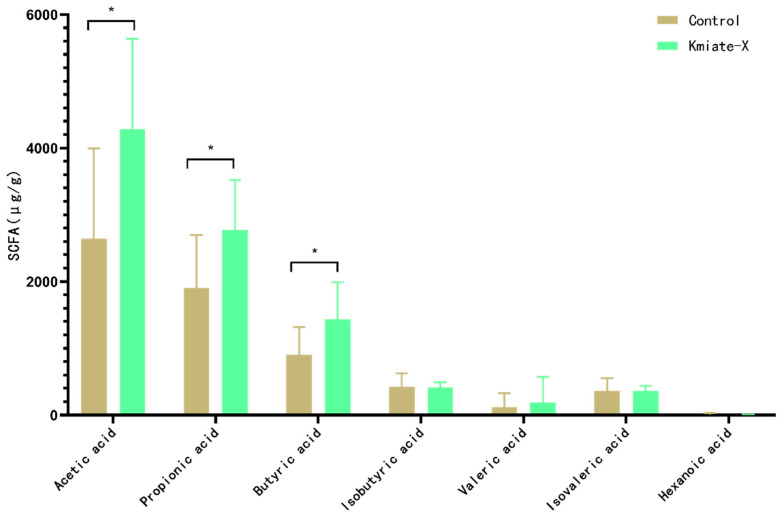
Effects of *Enterococcus faecium* Kimate-X on the content of short-chain fatty acids (SCFAs). Fecal samples were analyzed by GC–MS to quantify the main SCFAs. Bars show mean ± SD concentrations of acetate, propionate, butyrate, valerate, caproate, and heptanoate (n = 8 per group). Asterisks indicate statistically significant differences (* *p* < 0.05) versus the Control, determined by Student’s *t*-test.

Figure 5 shows the differences in SCFA content between the Control and Kimate-X group. The study found that Kimate-X treatment significantly increased the levels of acetate compared to the Control (*p* < 0.05). Propionate levels were also significantly higher in the Kimate-X group than in the Control (*p* < 0.05). Additionally, as an important indicator of gut health, butyrate content was significantly increased in the Kimate-X group (*p* < 0.05). However, there were no significant differences in the contents of valerate, caproate, and heptanoate between the two groups.

## 4. Discussion

Recent studies have increasingly shown that stress can impair the intestinal barrier, disrupt gut microbiota balance, and exacerbate inflammation, thereby intensifying the overall stress response [15,16,17,18]. At the same time, accumulating evidence indicates that the gut microbiota plays a crucial role in regulating stress responses [19,20]. The bidirectional gut–brain axis provides communication between the gut and the central nervous system via neural (especially the vagus nerve), endocrine, and immune pathways [21]. Through this axis, gut microbes can influence brain function by transmitting signals (e.g., microbial metabolites and neurotransmitters) that modulate the host’s stress-related physiology. Indeed, certain probiotics can fortify the gut barrier, reduce inflammatory responses, produce beneficial metabolites such as short-chain fatty acids (SCFAs), and even influence neurotransmitter production (for example, increasing GABA or serotonin levels), thereby mitigating stress effects [22,23]. Probiotic-based interventions have thus gained attention as a promising strategy for managing stress in animals [24]. In this study, we evaluated the efficacy of the novel probiotic *Enterococcus faecium* Kimate-X in alleviating transportation stress in dogs and explored its underlying mechanisms.

Our findings demonstrate that Kimate-X exerts significant antioxidant and anti-inflammatory effects in vitro. In LPS-challenged RAW 264.7 macrophages, Kimate-X treatment markedly increased the activities of antioxidant enzymes (SOD, CAT, GSH-PX) and lowered levels of the oxidative stress marker MDA and the inflammatory cytokine TNF-α compared to untreated cells. These results indicate that Kimate-X can counteract oxidative and inflammatory stress by bolstering the cellular antioxidant defense system and inhibiting pro-inflammatory pathways. This is consistent with previous reports that probiotics ameliorate host oxidative stress and inflammation; for example, YiTing et al. showed that probiotic supplementation attenuated inflammatory responses by suppressing NF-κB and MAPK signaling [25]. The antioxidant effects of Kimate-X may also be partly due to its metabolites (such as SCFAs), which have known antioxidant and anti-inflammatory properties [26,27].

In vivo, Kimate-X supplementation similarly alleviated physiological stress markers in dogs subjected to transport. Dogs receiving Kimate-X showed enhanced antioxidant status (significantly higher SOD and GSH-PX activities) and reduced oxidative damage and inflammation (significantly lower MDA and TNF-α levels) after transport compared to Control dogs. These outcomes suggest that Kimate-X effectively attenuated transport-induced oxidative stress and inflammatory responses by strengthening the host’s antioxidant defenses and modulating immune activation. Notably, our results align with other studies supporting probiotics as modulators of stress: for instance, a Lactobacillus-based probiotic was reported to reduce stress-induced cortisol levels by influencing the gut–brain axis [28]. Consistently, we observed that Kimate-X-treated dogs had significantly lower serum cortisol post-transport than Controls. Cortisol is a crucial stress indicator (its level typically correlates with stress intensity) [29], and the reduction in cortisol further confirms the anxiety-attenuating effect of Kimate-X. This effect is likely mediated by Kimate-X’s influence on the hypothalamic–pituitary–adrenal (HPA) axis via gut–brain signaling, as seen in other probiotic interventions [30].

Kimate-X supplementation also induced pronounced changes in the gut microbiota of the dogs. Treated dogs exhibited significantly higher microbial diversity (Shannon and Simpson indices) than Controls, indicating that Kimate-X enriched the gut microbiota’s richness and evenness. Greater microbial diversity is generally associated with improved gut health and resilience to stress [31]. Community composition analyses (PCoA) showed a clear separation between the Kimate-X and Control groups, confirming that Kimate-X substantially remodeled the gut microbiome structure [32]. At the taxonomic level, certain commensals were differentially affected: for example, fiber-fermenting genera such as *Prevotella* (copri clade A) and *Streptococcus* (*S. lutetiensis*) had higher relative abundance in the Kimate-X group, whereas a Lactobacillus species (*L. johnsonii*) was more abundant in Controls [33]. These shifts suggest that Kimate-X creates a gut environment favoring SCFA-producing and potentially beneficial microbes, even if some typical probiotic bacteria (e.g., lactobacilli) do not increase in abundance. Such changes in microbiota composition are in line with the theory that probiotics improve host well-being by restoring a healthy microbial balance [34].

Metagenomic functional analyses further revealed that Kimate-X influenced the metabolic capacity of the gut microbiome. Dogs in the Kimate-X group showed higher enrichment of pathways related to biotin metabolism, whereas Control dogs had a greater representation of starch and sucrose metabolism. Biotin is an essential cofactor in various metabolic processes, and increased microbial biotin metabolism may benefit the host by enhancing nutrient synthesis and gut mucosal health [35]. In contrast, the lower emphasis on simple carbohydrate metabolism in Kimate-X-treated dogs could reflect a shift in the microbiota toward more complex fiber utilization and efficient energy use. Consistently, we found that fecal SCFA levels (acetate, propionate, and butyrate) were significantly elevated in the Kimate-X group compared to Controls. SCFAs serve as energy sources for colonocytes and have broad anti-inflammatory and immunomodulatory effects; for example, butyrate can inhibit NF-κB signaling to reduce intestinal inflammation [36,37]. Thus, Kimate-X’s ability to boost SCFA production and key metabolic pathways is likely an important contributor to its protective effect against stress.

Compared to more commonly used probiotics such as *Lactobacillus* and *Bifidobacterium* spp., Kimate-X exhibits both similar and distinct anti-stress mechanisms. Lactobacillus and Bifidobacterium strains are well known to alleviate stress-related symptoms by strengthening the gut barrier, modulating immune responses, and even producing calming neurotransmitters like GABA [38]. Kimate-X appears to share these general pathways—evidenced by its reduction in inflammation and cortisol—while uniquely reshaping the gut microbiota composition. Notably, Kimate-X increased beneficial fermentative bacteria and SCFA output without necessarily increasing the abundance of *Lactobacillus* or *Bifidobacterium* in the gut. This suggests that different probiotic species can achieve stress relief through convergent physiological outcomes (e.g., HPA axis modulation and reduced inflammation) but via distinct interactions with the host microbiome. Further comparative studies would be valuable to determine how Kimate-X’s efficacy stacks up against traditional probiotics and whether their effects could be complementary.

It is also important to recognize the biological variability in gut microbiota responses among individual hosts. In our study, using juvenile male Beagle dogs helped minimize variability due to age, sex, and baseline microbiota differences, thereby isolating the effects of Kimate-X [39,40]. However, in broader populations of dogs, differences in breed, diet, and environment could influence the degree of response to this probiotic. Future research should consider such variability and evaluate Kimate-X in diverse groups and real-world settings to ensure consistency and generalizability of its anti-stress benefits. Nonetheless, the robust effects observed here suggest promising practical applications for Kimate-X in canine health. For example, this strain could be incorporated into pet foods or supplements as a natural strategy to help dogs cope with common stressors (e.g., transportation, kenneling, or veterinary visits). Veterinary clinics and pet caretakers might use Kimate-X as a probiotic intervention to reduce stress-related gastrointestinal upset or anxiety in dogs, offering an alternative or adjunct to pharmacological treatments. In environments like animal hospitals or boarding kennels, routine Kimate-X supplementation may improve animal welfare by diminishing stress-induced health issues.

Overall, our findings provide a foundation for translating Kimate-X into clinical practice as a novel probiotic approach for stress management in dogs, though further field trials are warranted to confirm its efficacy and safety in various settings.

## Data Availability

The datasets generated and/or analyzed during the current study are available from the corresponding author upon reasonable request.

## Supplementary Materials

Table S1. Taxonomic classification and relative abundance of bacterial species identified from canine fecal samples. Taxonomic ranks include Kingdom, Phylum, Class, Order, Family, Genus, and Species. Relative abundance values are provided for each individual sample from the control group (Con1–Con8) and the probiotic intervention group (Kmiate-X1–Kmiate-X8), along with the overall average abundance. The following supporting information can be downloaded at https://www.mdpi.com/article/10.3390/vetsci12050412/s1.
